# Drug screening of α‐amylase inhibitors as candidates for treating diabetes

**DOI:** 10.1111/jcmm.17831

**Published:** 2023-07-04

**Authors:** Meryem Alp, Alechania Misturini, German Sastre, Maria Gálvez‐Llompart

**Affiliations:** ^1^ Department of Physics Gazi University Ankara Turkey; ^2^ Instituto de Tecnología Química, Universitat Politécnica de Valéncia Valencia Spain; ^3^ Department of Preventive Medicine and Public Health, Food Science, Toxicology and Forensic Medicine, Faculty of Pharmacy University of Valencia Valencia Spain; ^4^ Department of Physical Chemistry, Molecular Topology and Drug Design Unit University of Valencia Valencia Spain

**Keywords:** acarbose, database screening, docking, molecular dynamics, protein binding, type‐2 diabetes

## Abstract

In the present study, the identification of potential α‐amylase inhibitors is explored as a potential strategy for treating type‐2 diabetes mellitus. A computationally driven approach using molecular docking was employed to search for new α‐amylase inhibitors. The interactions of potential drugs with the enzyme's active site were investigated and compared with the contacts established by acarbose (a reference drug for α‐amylase inhibition) in the crystallographic structure 1B2Y. For this active site characterization, both molecular docking and molecular dynamics simulations were performed, and the residues involved in the α‐amylase–acarbose complex were considered to analyse the potential drug's interaction with the enzyme. Two potential α‐amylase inhibitors (AN‐153I105594 and AN‐153I104845) have been selected following this computational strategy. Both compounds established a large number of interactions with key binding site α‐amylase amino acids and obtained a comparable docking score concerning the reference drug (acarbose). Aiming to further analyse candidates' properties, their ADME (absorption, distribution, metabolism, excretion) parameters, druglikeness, organ toxicity, toxicological endpoints and median lethal dose (LD_50_) were estimated. Overall estimations are promising for both candidates, and in silico toxicity predictions suggest that a low toxicity should be expected.

## 1 INTRODUCTION

Diabetes mellitus is one of the most common non‐communicable metabolic disorders categorized by type‐1 or insulin‐dependent diabetes, type‐2 or non‐insulin dependent diabetes mellitus and gestational diabetes mellitus.^1^ Among these, type‐2 diabetes is a pandemic and has affected almost 200 million people globally, and this figure is likely to rise gradually to 642 million by 2040. It happens when the pancreas stops using insulin effectively, which means the body's cells lose the ability to respond to insulin's efforts to drive glucose into the cells, a condition called insulin resistance leads to a type of metabolic disorder in which the patient has an increased level of sugar (glucose) in blood, which can lead to potential complications that include blindness, heart attacks, strokes, kidney damage, lower limb amputation and nerve damage. The management of blood glucose in patients affected by diabetes mellitus is complex because of personal factors, such as exercise and diet, and the non‐specific mode of action associated with oral antihyperglycemic.^2^

In the present scenario, enzyme inhibitors can be explored as alternative targets due to their high degree of substrate specificity. Amylase is a digestive enzyme, mainly produced by the salivary glands and pancreas, serving both endocrine and exocrine functions. Among the different types of amylase enzymes, one major category of the enzyme is pancreatic α‐amylase, which is a calcium metalloenzyme that aids digestion by breaking down polysaccharide molecules into smaller ones, such as glucose and maltose. In addition, the enzyme causes postprandial hyperglycemia and blood glucose levels to rise. By performing the act of inhibiting α‐amylase enzyme, it helps in reducing hyperglycemia, obesity and problems such as overweight conditions by reducing postprandial levels of glucose.

Alpha‐amylase is the major form of amylase found in humans and other mammals. It is an enzyme that hydrolyses α‐bonds of large, α‐linked polysaccharides, such as starch and glycogen, yielding shorter chains thereof, dextrins and maltose.^3^ As α‐amylase is an important target for the treatment of diabetes mellitus and the development of new drugs, scientists are showing great interest in this enzyme.^4^ In this regard, 3D computer‐aided drug design methods, such as molecular docking and molecular dynamics (MD), take into account ligand‐receptor simulation systems.^5^ Molecular docking analysis models the interaction between a molecule and a protein at the atomic level. This information permits us to characterize the behaviour of molecules in the binding sites of target proteins.^6^

An essential computational tool for analysing the time evolution of biological structures is MD. The stability and conformation of macromolecules, as well as their host‐guest complexes with smaller compounds (i.e. drugs), can be better understood by MD simulations. Moreover, the role of molecular flexibility, solvent and counter‐ions can be assessed, as they are not taken into account in the rigid molecular docking calculations.

In the present study, receptor‐based methods are combined in order to design new, potential α‐amylase inhibitors for the control and reduction of blood sugar levels and the treatment of type II diabetes.

## 2 COMPUTATIONAL MODELS AND METHODS

### 2.1 Computational strategy to identify novel α‐amylase inhibitors

In order to identify novel α‐amylase inhibitors for the treatment of type‐2 diabetes mellitus, we depicted a structure‐based drug design strategy to select potential candidates to inhibit α‐amylase. For this purpose, pancreatic α‐amylase co‐crystallized with the reference antidiabetic drug acarbose was retrieved from the Protein Data Bank (PDB: 1B2Y).^7^ The steps followed were:

1. *Virtual high‐throughput screening*: a molecular docking screening was performed in order to identify potential α‐amylase inhibitors. Three different commercial databases were considered: Academic and Natural Compounds collection (from Specs)^8^ and Natprod collection (from Discovery Systems, Inc.).^9^ From over 380,000 molecules, 16 potential α‐amylase inhibitors were selected.
2. *Validation of the binding site*: the magnitude and nature of interaction with the reference drug was investigated. From blind and site‐specific docking, as well as MD simulations, enzyme residues interacting with the acarbose could be tracked, and used as references in the next step. The drug stability in the binding pocked could also be verified. Acarbose was selected as a reference antidiabetic drug in order to check if there is a similarity between the experimental location of α‐amylase and the calculated location of the candidate inhibitors.
3. *Candidate drugs interaction with* α‐amylase: a blind and site‐specific docking analysis was performed in order to check whether these 16 potential α‐amylase inhibitors will interact preferentially with the reported binding site with therapeutic activity.

For the tasks above, molecular docking and MD calculations were performed as described below.

### 2.2 Molecular docking

Molecular docking studies play a major role in determining whether millions of synthesized compounds are effective drug substances. The calculation of the interaction of a chemical compound on a protein structure allows the prediction of different binding modes. Since the docking process brings powerful insights for explaining the enzyme‐ligand relationship, it plays an important role in enzyme inhibition studies.^6^ For the present study, in order to predict the potential activity of novel α‐amylase inhibitors, the structure of human pancreatic alpha‐amylase in complex with the carbohydrate inhibitor acarbose (PDB: 1B2Y)^7^ was retrieved from the RCSB PDB.^10^ This model provides a good balance between 3D protein structure resolution (3.2 Å) and accurate information (*R*‐value free: 0.217) about the reference drug (acarbose) binding site.

Docking calculations were performed using AutoDockVina^11^ and Schrödinger^12^ software suite molecular modelling packages (version 2021–3), using default parameters unless otherwise reported.

Two types of docking calculations were performed: site‐specific docking and blind docking. Site‐specific docking focuses on a reduced region of the protein that is selected as the preferred binding active site, so that all Monte Carlo moves are centred in this region. Here, the binding site was defined by the crystallographic position of the co‐crystallized antidiabetic drug (acarbose). Blind docking considers the whole protein as potential sites for binding, and allows obtaining, as a result, a shortlist (called ‘modes’) of the potential active sites within the protein for the ligand. If some of these modes correspond to the active site of known drugs with demonstrated therapeutic action, it is an additional indication that the molecule being tested could be a good candidate with the same potential therapeutic action.

Therefore, although knowing the catalytic site of an enzyme is crucial, it is still important to perform a parallel blind docking or unguided analysis to study other potential interactions of the compound with other binding sites of the target protein. This information provides insight into the specificity of the drug with respect to the pharmacological target. By employing this strategy, we can evaluate if the most favourable binding site of potential drug candidates is within the protein's active site. Blind docking is, therefore, an excellent benchmark to test the accuracy of our computational methods. Since the docking site is known, blind docking should render the active site as one of the most energetically favourable for binding, and this condition has been satisfied by acarbose.

Moreover, a vital component of the molecular docking pipeline is the scoring function, as it determines the fitness of sampled poses (modes).^13^ The scoring function is used to evaluate the binding affinity of the ligand to the protein, and it is based on various factors such as van der Waals interactions, hydrogen bonding, electrostatic interactions and solvation effects.^14^ Albeit the score value obtained from molecular docking is not always accurate, it is still a useful tool for predicting and ranking the binding affinity of different ligands to a macromolecule, being widely used in drug discovery studies.

In addition to all these important and useful features of molecular docking studies, they should be supported by MD. The molecule, which is buried inside the protein structure, is also interacting with the solvent. The thermal effects on the kinetic energy of the molecule and on the protein conformation could lead to an activated jump of the molecule between different interaction sites that can be studied using MD calculations.

### 2.3 Molecular dynamics simulations

According to the literature, there is good agreement between computational and experimental measurements of macromolecular dynamics.^15^ Since receptor dynamics and flexibility play a key role in their interaction with candidate drugs, MD simulations can be a powerful tool for drug discovery studies. To complement the docking study and further evaluate the changes in enzyme and drug conformations, MD simulations were performed with *pmemd*.*cuda* software from the AMBER16 simulation package.^16^ The α‐amylase was described by the ff99SB‐ILDN force field,^17^ since it presents good agreement with experiments when in conjunction with TIP4P‐Ew^18^ water model.^19^, ^20^ General Amber Force Field^21^ was considered to model drug molecules, as it is compatible with Amber force fields such as f99.^21^ An all atom modelling of the system was considered, where hydrogen atoms for the macromolecule and drug were added with *pdb4amber* program, from Ambertools package. Enzyme‐ligand complexes were solvated with TIP4P‐Ew water molecules, considering a solvation shell of at least 10.0 Å with respect to any solute atom. Na^+^ and Cl^−^ ions were added to the simulation box to neutralize the system and to simulate a saline solution (0.15 M), as previously reported in the literature.^22^ AM1‐BCC partial charges^23^, ^24^ were calculated for the enzyme‐acarbose complexes.

First, energy minimization of the simulation boxes was performed, followed by temperature and pressure equilibration of the systems. The temperature was gradually increased to 298.15 K during an MD simulation of 100 ps at the NVT ensemble. Then, the thermalized system was simulated in the NPT ensemble for 250 ps and at 1 bar. Temperature and pressure control was ensured by Langevin thermostat^25^ and Monte Carlo barostat^26^ for orthogonal boxes, respectively. Next, the production run was performed, with trajectories being computed over 135 ns. Every MD simulation considered a time step of 2.0 fs, which was possible due to constraints applied to bonds containing hydrogen atoms, by the SHAKE algorithm.^27^ To avoid finite‐size effects, periodic boundary conditions were considered. Finally, a cutoff of 9.0 Å was set for the nonbonded interactions, while electrostatic interactions were treated with particle mesh Ewald^28^ method.

The hydrogen bonds (HB) established during the simulations, mass‐weighted root mean square deviation (RMSD), mass‐weighted radius of gyration (*R*_g_), gyration tensor and 3D histogram of drug conformations were computed from trajectories by *cpptraj* ^29^ program, available in the AmberTools. Additional analyses are included in Section S2 of Supplementary Information (SI).

### 2.4 Computational toxicology study

In current drug development studies, the potential of a novel chemical is commonly evaluated initially through virtual tools.^30^ The aim of this analysis is to predict organ toxicity, toxicological endpoints, median lethal dose (LD_50_), druglikeness and ADME (absorption, distribution, metabolism, excretion) parameters of the selected molecules. The enzyme–drug complexes were screened using in‐silico Pre‐ADMET software^31^ and SwissADME^32^ to estimate their overall ADME properties and toxicity hazards. Furthermore, ProTox‐II software^33^ was used to estimate the organ toxicities and toxicological end points of molecules and their LD_50_ (median LD_50_, the amount which causes the death of 50% of a group of test animals) values.

## 3 RESULTS AND DISCUSSION

We outline here the strategy to identify novel α‐amylase inhibitors with antidiabetic activity by employing two different approaches: molecular docking studies and MD. Initially, virtual high‐throughput screening was conducted to generate a potential list of α‐amylase inhibitors. Subsequently, the binding site for α‐amylase antidiabetic activity was validated. Once the active binding site has been validated, potential antidiabetic compounds were docked (blind and site‐specific docking) with α‐amylase so that the amino acid interactions and docking score (DS) values were determined. Only molecules with both low DS and the same amino acid interaction pattern with α‐amylase as acarbose will be selected as molecules with potential therapeutic antidiabetic activity.

Then, MD simulations were performed in order to identify and assess the stability of the ligand‐α‐amylase interaction determined by molecular docking.

### 3.1 Virtual screening of potential α‐amylase inhibitors

A site‐specific molecular docking study was performed for the reference α‐amylase inhibitor (acarbose) as well as over 380,000 compounds from three different commercial databases. The Schrödinger software,^12^ employing the OPLS3e force field,^34^ allows performing high‐performance ligand‐receptor docking to accelerate structure‐based drug design. This is made possible by Schrödinger because it offers different ranges of speed/accuracy options from the HTVS (high‐throughput virtual screening) mode to the XP (extra precision) mode, where a more extensive sampling and advanced scoring is performed. The receptor grid for this HTVS was generated considering the position of the co‐crystallized reference antidiabetic drug acarbose in the α‐amylase reported structure (PDB: 1B2Y). Sixteen compounds (Table 1) were selected as potential α‐amylase inhibitors as their DS values (Figure S1) were lower than the selected criterion of −7.95 kcal/mol, which corresponded to the DS value of acarbose (reference drug for the treatment of type 2 diabetes mellitus).

**TABLE 1:** High‐throughput site‐specific docking score (DS) values (Schrödinger software) of potential α‐amylase inhibitors across three databases of compounds. For reference, the value of acarbose is also included.

Compound	DS	Database
Acarbose	−7.95	Reference
Hematoporphyrin	−9.36	Discovery Systems, Inc.
Octopamine	−9.01	Discovery Systems, Inc.
AN‐153I105594	−8.95	Specs
Kynuramine	−8.83	Discovery Systems, Inc.
Diptrobin A	−8.69	Discovery Systems, Inc.
AN‐153I104161	−8.63	Specs
AN‐153I100678	−8.62	Specs
AN‐153I101592	−8.53	Specs
AN‐153I103354	−8.43	Specs
Hederacoside C	−8.40	Discovery Systems, Inc.
Danuorobicin	−8.33	Discovery Systems, Inc.
AN‐153KC12612	−8.23	Specs
AN‐153I104845	−8.15	Specs
Geneticin	−8.11	Discovery Systems, Inc.
AN‐153I103073	−8.10	Specs
AN‐153I100720	−8.02	Specs

### 3.2 Αlpha‐amylase binding site validation: Blind docking versus site‐specific docking

The structure of the α‐amylase co‐crystallized with the reference antidiabetic drug acarbose (PDB: 1B2Y) contains specific amino acids that interact preferentially with acarbose and are related to its pharmacologic activity. A heavy benchmark for the software should be to test if a blind docking calculation of acarbose in α‐amylase is able to find the specific site of interaction as found in the crystallized structure. For this task, we have used AutodockVina. Water and other additional molecules present in the crystallographic structure were removed from α‐amylase for the calculations. The enzyme's polar hydrogens were explicitly considered, and Kollman partial charges^35^ were calculated. For the drug candidates, Gasteiger partial charges^36^ were defined and non‐polar hydrogen atoms were added. Finally, the complexes were subsequently analysed and ranked based on their binding affinity. The interaction profiles were utilized to evaluate the interactions between the protein and the compounds. The history files for the selected docking poses were stored and visualized using Biovia Discovery Studio Visualizer software^37^ (version 21.1).

**FIGURE 1:**
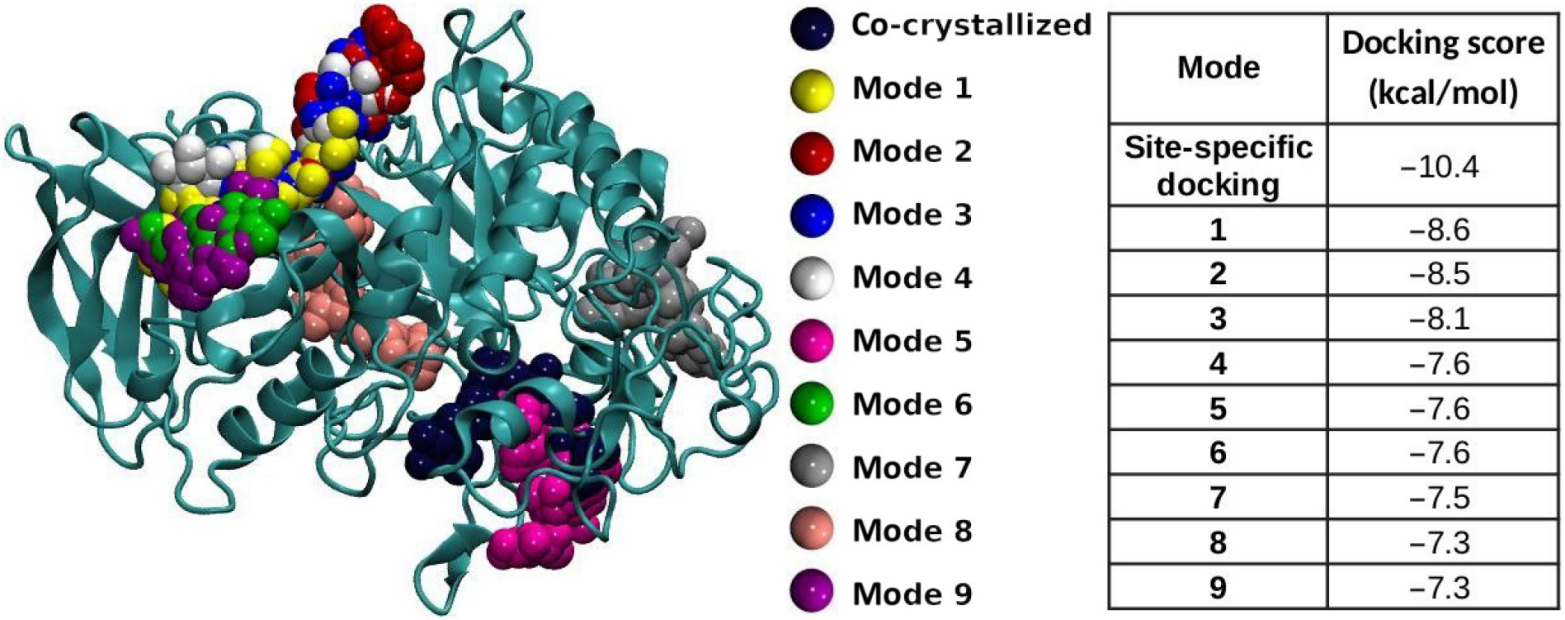
The most stable locations (modes, left) of acarbose (antidiabetic reference drug) in complex with α‐amylase from blind docking studio performed with AutodockVina. Acarbose co‐crystallized position (what we also call ‘active site’) is also shown for the sake of comparison. Acarbose blind docking score (DS) values on α‐amylase using AutoDockVina (right). For reference, the site‐specific DS is also included. ‘mode 5’ corresponds, very approximately, to the same location as that for the site‐specific docking, which in turn corresponds to the active site experimentally found (PDB: 1B2Y).^7^

The results are shown in Figure 1. In the calculations, only the nine most stable locations will be selected, called from ‘mode 1’ to ‘mode 9’, according to the notation employed by AutoDock‐Vina software, with ‘mode 1’ being the most stable and the others corresponding to increasingly weaker compound‐protein interactions. ‘Mode 5’, among the most stable, is reasonably close to the experimental location of acarbose.

Considering the large number of possible binding sites throughout the entire protein, ‘mode 5’, among the most stable, is still reasonably close to the experimental location of acarbose. It is important to note that the use of more accurate methods to a high‐throughput screening has a prohibitive computational cost. For instance, a direct application of quantum mechanics to protein systems is unfeasible due to the large molecular size of proteins,^38^ and as a consequence, there is no general quantum mechanics‐based method for simulating protein dynamics. On the other hand, even if considering a well parameterized force field, the utilization of MD simulations for a high‐throughput screening is extremely expensive computationally, specially from the need of enhanced sampling methods for a proper simulation of the enzyme conformational space. Therefore, we have to come down to the limited accuracy of molecular docking simulations using force fields,^39^ in order to explore a wide variety of new candidate drugs.

**FIGURE 2:**
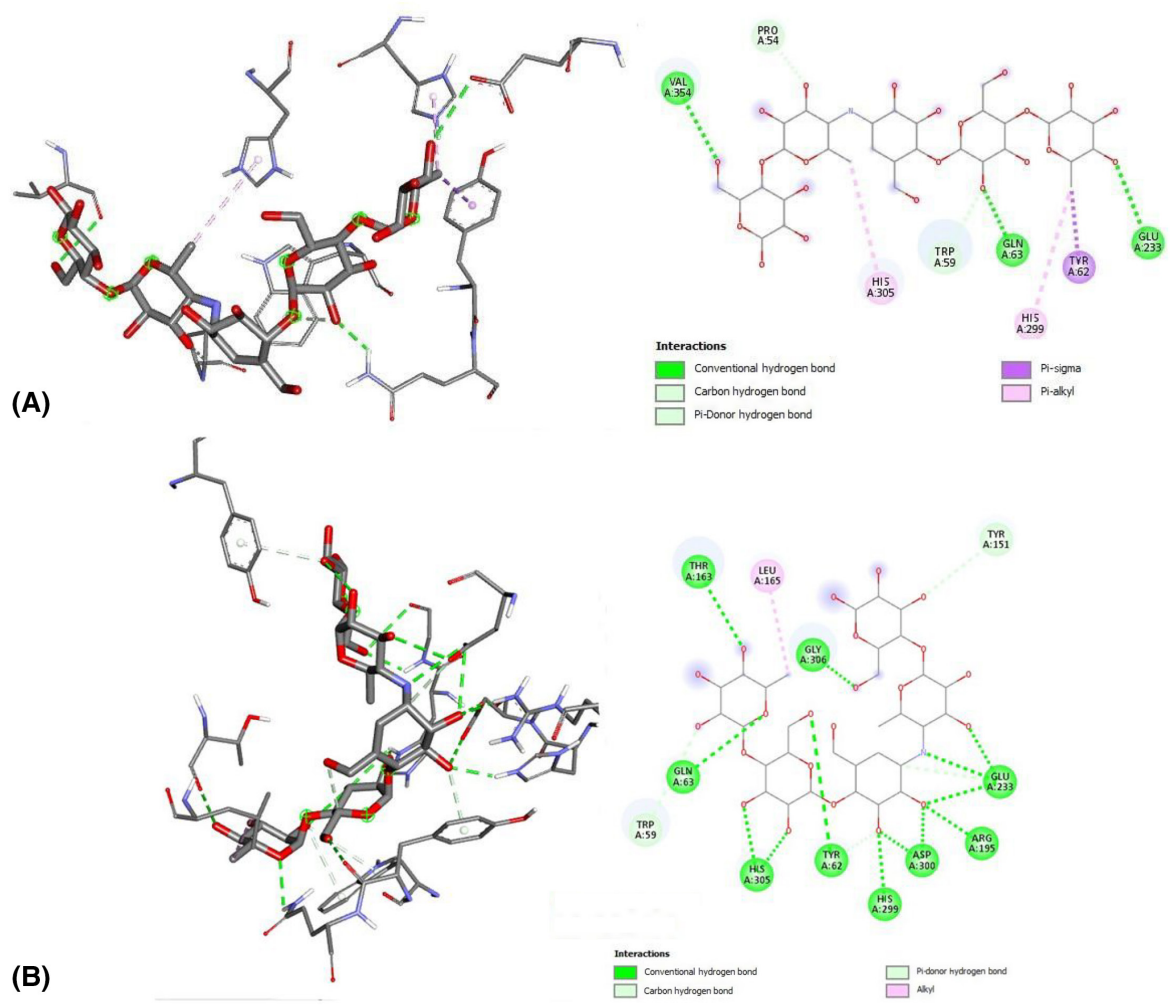
(A) Acarbose interactions with α‐amylase as calculated from blind docking (mode 5). (B) Acarbose interaction with α‐amylase as calculated from site‐specific docking (mode 1, see ‘site‐specific’ in Figure 1, right), resulting in a location very close to the crystallographically reported.^7^

An analysis of specific interactions between α‐amylase and acarbose in mode 5 (blind docking) is shown in Figure 2A. Docking of the compounds to the enzyme revealed a network of non‐covalent interactions such as hydrogen bond (HB), pi‐sigma, pi‐pi (T‐shaped and stacked conformations) and pi‐alkyl interactions. Mode 5 of acarbose is close to the experimental location that defines the active site with the therapeutic activity that should be the target for potential new antidiabetic drugs. Site‐specific docking, by giving a location closer to the crystallized location (Figure 2B), allows for establishing a more accurate estimation of the relevant interactions between α‐amylase and acarbose (details are given in Table S2), and they are included as a reference in Table 2. A comparison with other compounds will allow an assessment of their potential therapeutic activity.

**TABLE 2:** Selected results from site‐specific and blind docking of potential antidiabetic compounds with α‐amylase using AutoDock Vina.

Compound	NBC	Site‐specific docking			Blind docking		
		DS	Amino acid interaction	NMAA	DS	Amino acid interaction	NMAA
Acarbose	‐	−10.4	*HB*: **ASP‐300, ARG‐195, GLY‐306, GLU‐233, GLN‐63, HIS‐299, HIS‐305, TYR‐62, THR‐163, TYR‐151, TRP‐59** *Alkyl*: **LEU‐165**	12	−8.6	*HB*: ARG‐252, ARG‐421, GLY‐403, TRP‐280, HIS‐331, PRO‐332, PRO‐405 *Alkyl*: PRO‐4	‐
Hederacoside C	8	−9.1	*HB*: **ASP‐300**, ASP‐356, **GLU‐233**, **GLY‐306**, GLY‐304, ASN‐352, TRP‐357, **HIS‐305** *Pi‐Alkyl*: **TRP‐59**, TRP‐58 *Alkyl*: **LEU‐165** *Pi‐Alkyl + Pi‐Sigma*: **TYR‐62**	7	−9.6	*HB*: GLU‐282, ARG‐421, ASP‐402 *Pi‐Alkyl*: PHE‐406 *Alkyl*: ARG‐398 *HB ± Alkyl*: PRO‐332	**0**
AN‐153I104845	8	−8.1	*Pi‐Alkyl*: ALA‐307, ALA‐198, LYS‐200 *HB*: **GLN‐63**, **GLY‐306, GLU‐233** *Pi‐Pi*: **TYR‐62** *Pi‐Alkyl* + *Pi‐Sigma*: ILE‐235 *Pi‐Pi + Pi‐Sulfur*: **HIS‐305** *Pi‐Sulfur + HB*: **TRP‐59** *Pi‐Pi + Pi‐Cation + Pi‐Donor*: HIS‐201	6	−7.4	*HB*: GLU‐240, **HIS‐305**, **THR‐163**, **ASP‐300**, ASP‐197 *Pi‐Sigma*: LEU‐162 *Pi‐Alkyl*: **LEU‐165** *Pi‐Sulfur + Pi‐Cation*: HIS‐201	4
AN‐153I105594	7	−10.8	*Pi‐Alkyl*: LEU‐162, **LEU‐165, HIS‐305, TYR‐151** *Alkyl*: LYS‐200 *Pi‐Pi*: **TYR‐62** *HB + Alkyl*: HIS‐201 *Alkyl + Pi‐Alkyl*: ILE‐235 *Pi‐Alkyl + Pi‐Pi*: **TRP‐59**	5	−10.3	*Pi‐Alkyl*: **TRP‐59**, HIS‐101, ALA‐198, LEU‐162, **HIS‐305** *Alkyl*: **LEU‐165** *Pi‐Sigma*: ILE‐235 *Pi‐Cation + Pi‐Pi*: HIS‐201 *Pi‐Alkyl + HB*: LYS‐200 *Pi‐Alkyl + Pi‐Pi*: **TYR‐151**	4
AN‐153I104161	7	−8.1	*Pi‐Alkyl*: ALA‐198, LYS‐200 *HB*: HIS‐101 *Pi‐Sigma*: ILE‐235 *Salt Bridge*: **GLU‐233** *Attractive Charge*: **ASP‐300** *Pi‐Pi*: **TYR‐62** *Pi‐Cation + Pi‐P*: HIS‐201 *HB + Attractive Charge + Pi‐Anion*: ASP‐197	3	−7.7	*Pi‐Alkyl*: LEU‐162, LYS‐200 *Pi‐Sigma*: ILE‐235 *Attractive Charge*: ASP‐197 *HB*: **GLY‐306** *Attractive Charge + Pi‐Anion*: **ASP‐300** *Pi‐Cation + Pi‐Pi*: HIS‐201 *Salt Bridge + Charge Attractive*: **GLU‐233**	3
Diprotin A	7	−7.0	*HB*: **HIS‐299**, ASP‐197, **GLU‐233** *Alkyl*: ALA‐198, LEU‐162 *Pi‐Alkyl*: **TRP‐59**, **HIS‐305**	4	−6.7	*HB*: **GLN‐63** *Pi‐Sigma*: **TRP‐59** *Alkyl*: ILE‐235, ALA‐198 LEU‐162 *Pi‐Alkyl*: **TYR‐62,** HIS‐201, **TRP‐59**	4
Daunorubicin	6	−9.3	*Pi‐Alkyl*: LYS‐200, ALA‐198 *HB*: **THR‐163** *Acceptor‐Acceptor*: **GLU‐233** *Pi‐Sigma*: LEU‐162 *HB + Pi‐Sigma*: **HIS‐305** *Alkyl + Pi‐Sigma*: ILE‐235 *Pi‐Cation + Pi‐Pi*: HIS‐201	3	−8.8	*HB*: **GLN‐63**, HIS‐101 *Alkyl*: LEU‐162 *Pi‐Alkyl + Pi‐Pi*: **TRP‐59**	2

*Note*: The compound location in site‐specific docking was defined by the experimental location of acarbose in α‐amylase (1B2Y). Relevant amino acid interactions with α‐amylase are specified, and those common to acarbose are highlighted in bold. Docking scores (DS) are in kcal/mol. Types of interactions are explained in Table S2. Number of modes in blind docking that correspond to the crystallographic bind‐pocket region (NBC), number of matching amino acids (NMAA) involved in interactions, considering acarbose site‐specific docking as reference.

**FIGURE 3:**
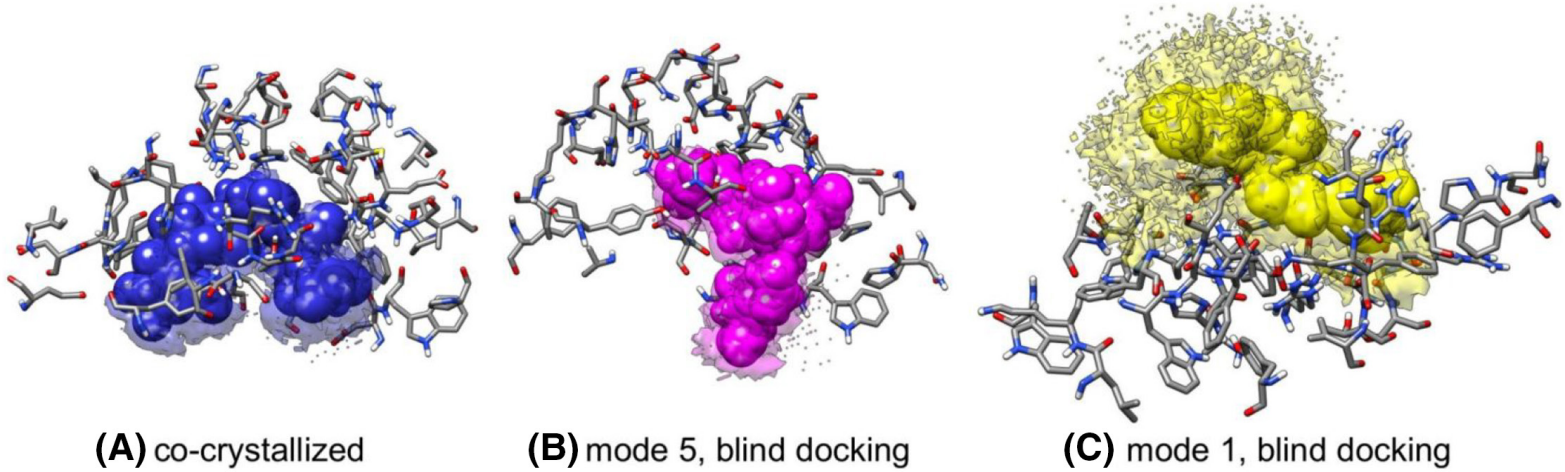
Average position of enzyme residues, up to 7.0 Å from acarbose, in licorice representation and average position of acarbose, in van der Waals representation. The transparent/dotted surfaces correspond to all acarbose conformations during the simulations, using as initial configuration the (A) co‐crystallized structure, (B) mode 5 from blind docking or (C) mode 1 from blind docking.

The partial overlap of the mode 5 configuration (from blind docking) in the actual binding site of the enzyme is confirmed by comparing the interacting residues depicted in Figures 2 and 3. Among the 12 residues interacting with the site‐specific docking geometry (Figure 2B; Table 2), six were detected in the mode 5 geometry (HIS‐305, TRP‐59, GLN‐63, HIS‐299, TYR‐62 and GLU‐233).

The analysis of amino acid binding site composition is chosen as a determining parameter to define similarity within binding sites. This approximation is based on the fact that amino acid composition is directly related to the specific geometry and chemical properties of the site, which determine the shape and volume of the suitable binding site, and define the electrostatic and hydrophobic properties of the pocket.

### 3.3 Αlpha‐amylase binding site validation: MD simulations of stability of α‐amylase‐acarbose complex

MD simulations were carried out in order to evaluate the stability of acarbose blind docking modes, when compared to the crystallographic structure. Furthermore, by taking into account a flexible system, solvated and with counter ions, the binding site and interactions established can be compared with the results from the previous section. Four simulations were considered: containing only the enzyme and the enzyme with acarbose (Figure S1A) at three different initial positions: experimental (PDB: 1BY2),^7^ mode 5 from blind docking (Figure 2A) and mode 1 from site‐specific docking (Figure 2B).

Movies of the MD simulations can be accessed by the QR codes in Figure S2 and they illustrate that, structurally, the three acarbose locations simulated do not lead to significant changes in the enzyme, as shown in Figure S4A by the small RMSD values of the enzyme (between 1.5 and 2.0 Å), which is analogous to the RMSD considering only the enzyme in solution. Likewise, the average radius of gyration gives very similar values, 23.1 ± 0.1 Å for the co‐crystallized geometry, and 23.3 ± 0.1 Å for mode 1 and mode 5 from blind docking and enzyme without drug.

**FIGURE 4:**
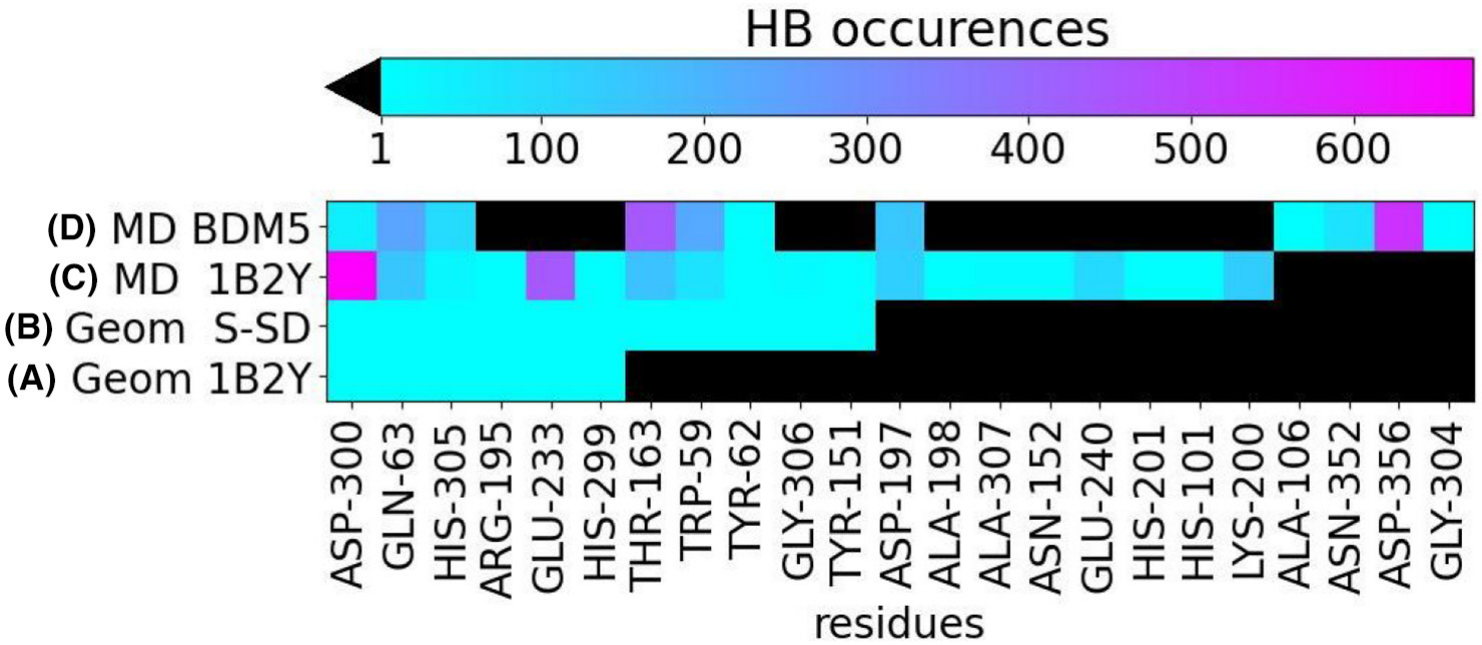
Hydrogen bonds established between enzyme and acarbose in: (A) co‐crystallized structure from PDB: 1B2Y; (B) Site‐Specific Docking; (C) the MD configurations obtained using the co‐crystallized structure as initial geometry (MD 1B2Y); (D) the MD configurations obtained using the Blind Docking mode 5 as initial geometry (MD BDM5). The number of occurrences is coloured in blue of increasing intensity and black when absent.

**FIGURE 5:**
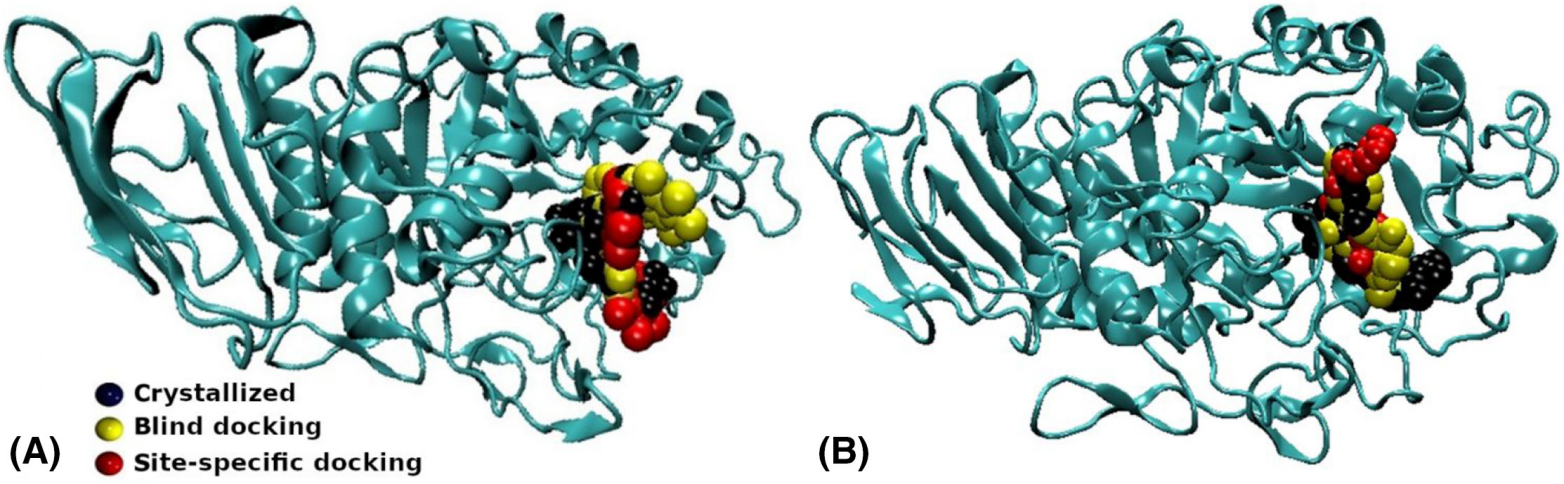
The most stable modes (best blind docking and site‐specific docking modes) of the (A) AN‐153I105594 and (B) AN‐153I104845 candidate and crystallized position of the reference drug acarbose in α‐amylase.

Concerning drug dynamics, acarbose stays bound to the enzyme throughout all sampling conformations, close to its initial configuration over the 135 ns simulation. While small changes in drug position were observed during the simulation of acarbose in its co‐crystallized geometry and blind docking mode 5 (Figures 4B, and 5A,B), mode 1 from blind docking presented wider mobility (Figures 4B, and 5C).

Acarbose geometry from 1B2Y crystallographic structure is composed by a disubstituted amine, containing a disaccharide and a trisaccharide group (Figure S1A). Although all acarbose chains are initially in contact with the enzyme surface in the initial configuration of the ‘mode 1’ simulation (Figure S3A), the disaccharide moiety presented considerable mobility, being completely solvated after the first 16 ns of simulation (Figure S3B), corresponding to the first significant change observed in the drug RMSD plot (yellow curve of Figure S4B). Nonetheless, the solvation of this disaccharide group is probably not the preferred state, as in the remaining time simulated, such a group re‐established interactions with the enzyme surface, but with conformations different from the original (Figure S3C).

Considering the co‐crystallized structure as a reference, by relaxing the acarbose structure in the α‐amylase binding pocket during the site‐specific docking, the number of HB increases from 6 to 11 (Figure 4A,B, respectively). When the co‐crystallized structure is simulated through MD (Figure 4C), a higher number of different HBs is observed (involving 19 residues), as a logical consequence of adding flexibility to the system. Even so, the main interactions (with colour tending to magenta in Figure 4C) correspond to the residues already accounted for in the site‐specific docking geometry (such as ASP‐300, GLU‐233, THR‐163, and GLN‐63). Thus, MD findings support the docking results that will be considered in the following section as the reference interactions.

Since acarbose therapeutic effect seems to suggest a relatively strong binding to α‐amylase, a stable active site can be expected from the MD simulations. This is in agreement with the binding free energy in solution obtained from the MD trajectories (Table S1, Section S2 of the SI). For the simulation starting in the co‐crystallized geometry, acarbose binding energy is the most stabilizing (−84.2 ± 7.4 kcal/mol), confirming that the preferential interaction site of acarbose is near the described active site. On the other hand, the acarbose binding free energies obtained from the simulations starting in mode 5 and mode 1 are −53.2 ± 5.6 kcal/mol and − 23.9 ± 10.3 kcal/mol, respectively. The lower stabilization of the latter mode is directly connected to the acarbose conformations adopted during this simulation, as previously discussed (Figure 3C). These results show the importance of relaxing the structure and considering the flexibility in order to understand the dynamic interactions between acarbose and α‐amylase.

In summary, although it is considerably important that various modes from blind docking analysis of a drug are found near the enzyme active site, these MD results reinforce the importance of using site‐specific docking as a criterion for the energy and the interactions between enzyme residues and candidate drugs.

### 3.4 Molecular docking analysis of potential antidiabetic compounds with α‐amylase: Blind and site‐specific docking approach

In this section, we will expand upon the molecular docking analysis of the potential α‐amylase inhibitors described in sections above. Using both blind (best mode #1) and site‐specific docking (using the experimental location 1B2Y), the main interactions of acarbose with the amino acid residues of α‐amylase have been determined. The calculations have been performed in AutoDockVina software, considering the AD4 scoring function, which uses potentials derived from early versions of AMBER force field.^40^ Consequently, the following results were described by models compatible with those used in the MD simulations. Amino acid interactions and DS with α‐amylase of the 16 potential antidiabetic compounds are reported in Table S3, which also contains the reference values for acarbose that were calculated previously.

If we analyse both DS and the relevant amino acid interactions (best‐performing candidates reported in Table 2, and a larger list of candidates in Table S3), two compounds stand out from the rest: AN‐153I105594 and AN‐153I104845. AN‐153I105594 presents a large number of interactions with the relevant amino acids of α‐amylase amino acids and a favourable DS (Table 2), both in site‐specific (−10.8 kcal/mol; LEU165, HIS305, TRP59, TYR151, TYR62) and blind docking (−10.3 kcal/mol; LEU165, HIS305, TRP59, TYR151). This is shown in Figure 5A. Seven of the nine modes whose geometry is analysed are very close to the targeted specific site (the active site of the protein). AN‐153I104845 also presents a large number of interactions with the relevant amino acids of α‐amylase amino acids and a favourable DS (Table 2), both in site‐specific (−8.1 kcal/mol; GLY306, HIS305, GLN63, TRP59, TYR62, GLU233) and blind docking (−7.4 kcal/mol; THR163, ASP300, HIS305, LEU165). This is shown in Figure 5B. Eight of the nine modes whose geometry is analysed are very close to the targeted specific site. This makes them good candidate drugs, since both molecules are likely to bind to the protein in the active site and interact with the same amino acid residues as those of acarbose.

Hederacoside C also presented a significantly stabilizing DS in the site‐specific (−9.1 kcal/mol; ASP‐300, GLU‐233, GLY‐306, HIS‐305, TRP‐59, LEU‐165, TYR‐62) and blind docking (−9.6 kcal/mol) calculations, and eight (out of nine) modes in the blind docking are close to the enzyme active site. However, from these eight binding modes close to the crystallographic bind pocket, the established interactions do not involve the same amino acids as those of acarbose, hence leading to a change in the nature of the enzyme‐drug interaction. In spite of this significant shortcoming, Hederacoside C could also be considered as a candidate drug for α‐amylase inhibition.

### 3.5 Computational toxicology study

In silico preliminary studies were performed with three enzyme–drug complexes (acarbose, AN‐153I105594 and AN‐153I104845) to estimate their toxicity, as well as the ADME properties. The computed druglikeness and ADME parameters are summarized in Table 3, which should be evaluated using Lipinski's rule of five. According to the Lipinski's rule of five, the molecular weight (MW) must be, ideally, less than 500, the Log *p* value must be less than five, and there must be fewer than 5 and 10 HB donors and acceptors, respectively. In addition, the total polar surface area (TPSA) is within acceptable bounds at 140 Å^2^. The investigation shows that the physicochemical characteristics of the reference molecule comply with Lipinski's criteria.^41^ Even though acarbose predicted values are not meeting some of the criteria, these calculations suggest that the candidate molecules show calculated properties within those expected for pharmacological agents.

**TABLE 3:** Calculated ADME/Tox and druglikeness properties of AN‐153I105594, AN‐153I104845 and acarbose.

ADME	AN‐153I105594	AN‐153I104845	Acarbose
MW (≤500 g/mol)	723.9	660.9	793.8
HBD (≤5)	2	3	14
HBA (≤10)	6	9	19
TPSA (≤140 Å^2^)	133	126	321
Log P (≤5)	3.0	4.2	0.6
GI absorption	Low	Low	Low
BBB (C.brain/C.blood)	0.078	0.113	0.027
Caco‐2 permeability (nm/s)	20.6	18.3	5.6
HIA (%)	98.6	95.6	0.0
Skin permeability (log Kp, cm/h)	−2.7	−2.3	−5.1
Toxicity prediction	AN‐153I105594	AN‐153I104845	Acarbose
Toxicity class	6	4	4
LD_50_ (mg/kg)	6350	500	1000
Organ toxicity (Hepatotoxicity)	Active	Inactive	Inactive
Carcinogenicity	Inactive	Inactive	Inactive
Immunotoxicity	Inactive	Inactive	Active
Mutagenicity	Inactive	Inactive	Inactive
Cytotoxicity	Inactive	Inactive	Inactive

*Note*: Toxicity class goes from 1 (toxic) to 6 (non‐toxic). Caco‐2 permeability is the permeability of compounds through a human intestinal epithelial cell barrier.

Abbreviations: ADME, absorption, distribution, metabolism, and excretion parameters; BBB, blood–brain barrier; GI, gastrointestinal absorption; HBA, number of hydrogen bonds acceptors; HBD, number of hydrogen bond donors; HIA, percentage of human intestinal absorption; LD_50_, median lethal dose; Log P, logarithm of the partition coefficient; MW, molecular weight; TPSA, total polar surface area.

The ADME study demonstrate that the values of Caco‐2 (colorectal cancer) cell permeability, blood–brain barrier penetration (BBB) and human intestinal absorption (HIA) are all well outside the limits of toxicity. BBB is typically a speed limiting factor for the presence of therapeutic drugs entering the brain. Therefore, it is very important to have delivery systems that can overcome this barrier for the treatment of brain‐based diseases such as Alzheimer disease, Parkinson disease and epilepsy.^42^ The permissible range of BBB penetration for an optimal drug candidate is 0.73–0.91^43^ and, although none of the two candidate drugs (AN‐153I105594 and AN‐153I104845) fall in this range, neither acarbose falls in this range. Hence, we do not expect particular problems of toxicity in this regard.

The amount of dosage that passes through the intestinal wall and into the portal vein after entering the digestive tract is typically used to calculate the human intestinal absorption (HIA).^44^ Drugs are divided into three classes according to the reported limit values for HIA. Poor absorption is defined as HIA ≤30%, while high absorption is defined as HIA ≥80% and ‘moderate’ absorption is defined as the residual range. The obtained HIA values indicate good oral absorption of the substances evaluated.

Permeability of compounds through a Caco‐2 cell is a widely used in vitro model to predict the absorption of drugs in the human intestine. Caco‐2 cells are derived from human colon adenocarcinoma and have the ability to differentiate into polarized cells that resemble the intestinal epithelium. This parameter is used to predict the fraction of an orally administered drug that will be absorbed into the systemic circulation.^45^ The Caco‐2 permeability levels for the selected candidates are low (up to 20 nm/s, Table 3) and hence within the acceptable range of 25–500 nm/s, while the reference drug presented an even lower Caco‐2 predicted value (5.6 nm/s).

The skin permeability (Kp) value is widely used to quantitatively describe the transport of molecules in the outermost layer of epidermal skin and indicate the significance of skin absorption. The standard range is −8.0 to −10.0 cm/h.^46^ In the event of accidental contact of the molecules with skin, a harmful effect may occur. Gastrointestinal absorption refers to the process by which a drug is absorbed from the gastrointestinal tract into the bloodstream. The range of reasonable values for gastrointestinal absorption may vary depending on the specific compound being studied. On the other hand, gastrointestinal absorption values reported in the literature for some compounds range from 60% to 0.01%, and a value below 25% is considered poor.^47^

Organ toxicities and toxicological end points of molecules and their LD50 (median LD_50_) were also assessed. LD50 is a standard measure used to determine the potential toxicity of a substance, where low values correspond to increased toxicity. Both candidates presented considerably high LD50 toxicity values (6350 mg/kg for AN‐153I105594 and 500 mg/kg for AN‐153I104845), indicating that no significant toxicity should be expected. Equally, the toxicity class values, from 1 (toxic) to 6 (non‐toxic), showed that the proposed molecules are non‐toxic (AN‐153I105594) or do not present acute toxicity (AN‐153I104845), similarly to acarbose predicted results. Overall toxicological predictions in Table 3 indicate that these compounds are noncarcinogenic in nature, and have no effect on mutagenicity and cytotoxicity. Some results of hepatotoxicity and immunotoxicity give activity, but the overall computationally predicted toxicological profile of these compounds (in particular, toxicity class and LD50) suggests AN‐153I105594 and AN‐153I104845 may not be toxic. Despite toxicity promising results, more sophisticated assays should be performed, and are beyond the scope of this work.

## 4 CONCLUSIONS

In order to explore the potential interactions between amino acids and α‐amylase, a combination of structure‐based drug design computational techniques, molecular docking and MD methodologies were utilized. By analysing the amino acid interactions observed between acarbose and the target enzyme (PDB: 1B2Y), key interactions relevant to developing antidiabetic pharmacological activity, similar to the reference drug, were identified.

To identify suitable candidates with potential antidiabetic activity, a high‐throughput docking screening involving over 380,000 compounds was performed. Based on DS and considering the co‐crystallized binding site of acarbose reported in the literature, 16 molecules were selected as potential α‐amylase inhibitors.

To investigate if some of these molecules could interact favourably with different binding sites within α‐amylase, a blind docking approach was employed. Among the 16 previously identified compounds, two (AN‐153I105594 and AN‐153I104845) were predicted to exhibit the most favourable binding at the active site.

Furthermore, the interactions between these two compounds and the amino acids of α‐amylase were found to be reasonably similar to those observed with acarbose. Therefore, these compounds can be considered potential α‐amylase inhibitors, similar to the reference drug, acarbose.

The selected candidates were also evaluated by their toxicity and ADME parameters employing in silico methods. Both candidate drugs met Lipinski's criteria, as they possess physicochemical properties expected for a medicament. Likewise, other desired properties, such as a high intestinal adsorption and a low toxicity, were predicted to AN‐153I105594 and AN‐153I104845 compounds, highlighting them as suitable candidates for further theoretical and experimental studies.

This work is sought to present a valuable computational strategy for the selection of novel α‐amylase inhibitors, as in silico studies are powerful tools to give useful insights and guide subsequent experimental work, such as in vitro α‐amylase inhibition essays.

## AUTHOR CONTRIBUTIONS

**Meryem Alp:** Data curation (equal); formal analysis (equal); investigation (equal); methodology (equal); software (equal). **Alechania Misturini:** Data curation (equal); formal analysis (equal); investigation (equal); methodology (equal); software (equal); validation (equal); visualization (equal); writing – original draft (equal); writing – review and editing (equal). **German Sastre:** Conceptualization (equal); formal analysis (equal); funding acquisition (equal); investigation (equal); methodology (equal); writing – original draft (lead); writing – review and editing (lead). **Maria Gálvez‐Llompart:** Conceptualization (equal); investigation (equal); methodology (equal); software (equal); supervision (equal); writing – original draft (lead); writing – review and editing (lead).

## FUNDING INFORMATION

The authors thank the Science and Technology Commission of Generalitat Valenciana (GVA) for funding through Prometeo 2021/077 and predoctoral fellowship GRISOLIAP/2019/084.

## CONFLICT OF INTEREST STATEMENT

The authors confirm that there are no conflicts of interest.

## Supporting information


Data S1:

## Data Availability

Data available in article Supporting Information. Chemical structure and names of the 16 compounds selected by the Virtual high‐throughput screening (Section S1), additional details concerning the molecular dynamics simulations such as movies, snapshots and binding free energy calculation (Table S1, Section S2), description of the different types of protein‐ligand interactions (Tables S2 and S3, Section S3). The datasets generated in the current study are available from the corresponding author on reasonable request.
