# Supplementary material for: Drug screening of α‐amylase inhibitors as candidates for treating diabetes
Source: J Cell Mol Med. 2023 Jul 4;27(15):2249–60. doi: 10.1111/jcmm.17831 (PMC10399542; doi:10.1111/jcmm.17831)
Supplement: Supplementary file 1 — Data S1: [file JCMM-27-2249-s001.pdf]

Supplementary Information

Drug screening of α-amylase inhibitors as candidates for treating diabetes

Meryem Alp[1], Alechania Misturini[2], German Sastre[2]*, Maria Gálvez-Llompart[3]

[1] Department of Physics, Gazi University, Ankara, Turkey, 06560

[2] Instituto de Tecnología Química, Universitat Politécnica de Valéncia, Avda. de los Naranjos s/n, 46022, Valencia, Spain; * e-mail: gsastre@itq.upv.es

[3] Molecular Topology and Drug Design Unit, Department of Physical Chemistry, University of Valencia, 46010 Valencia, Spain

Index

Section S1. Virtual screening of potential α-amylase inhibitors **S**2

Section S2. Molecular Dynamics simulations **S**6

Section S3. Interaction of candidate drugs with α-amylase **S**8

Section S1. Virtual screening of potential α-amylase inhibitors

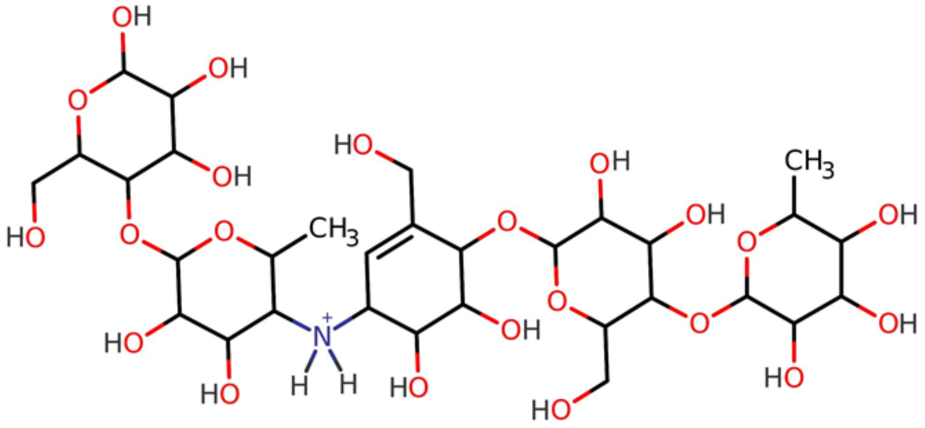

a) Acarbose: *alpha-D-quinovopyranose-(1-4)-alpha-D-glucopyranose-(1-4)-4,6-dideoxy-4-{[(1S,4R,5S,6S)-4,5,6-trihydroxy-3-(hydroxymethyl)cyclohex-2-en-1-yl]amino}-alpha-D-glucopyranose-(1-4)-beta-D-glucopyranose*

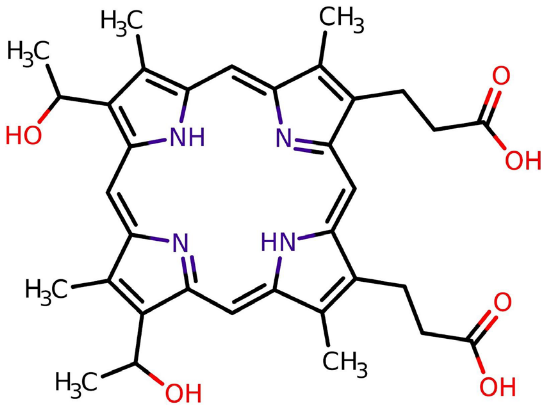

b) Hematoporphyrin:
3,3'-(7,12-bis(1-hydroxyethyl)-3,8,13,17-tetramethylporphyrin-2,18-diyl)dipropionic acid)

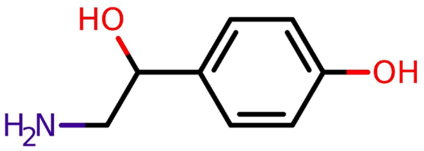

c) Octopamine:
4-(2-amino-1-hydroxyethyl)phenol

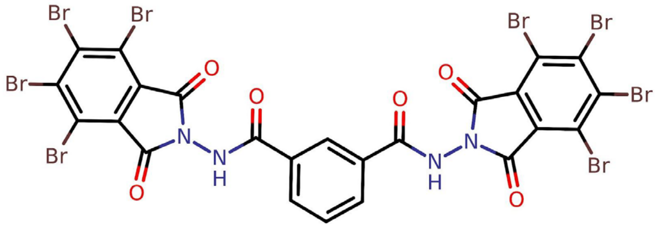

d) AN-153I105594:
N1,N3-bis(4,5,6,7-tetrabromo-1,3-dioxoisoindolin-2-yl)isophthalamide)

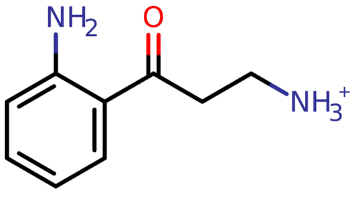

e) Kynuramine:
3-amino-1-(2-aminophenyl)propan-1-one

Figure S1. Chemical structure and names of the selected compounds by the Virtual high-throughput screening.

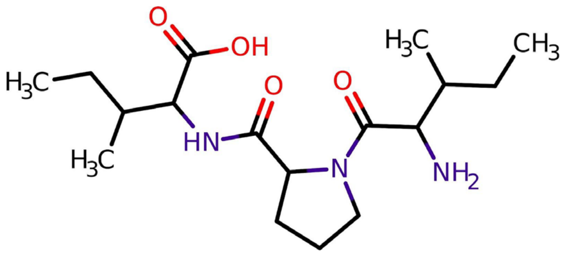

f) Diprotin A:
L-isoleucyl-L-prolyl-L-isoleucine

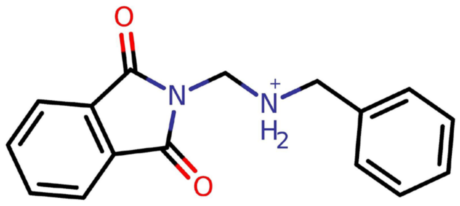

g) AN-153I104161:
2-((benzylamino)methyl)isoindoline-1,3-dione

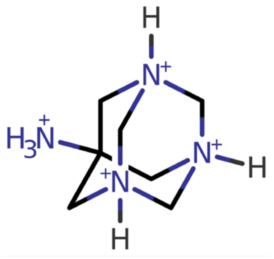

h) AN-153I100678
(1s,3s,5s)-1,3,5-triazaadamantan-7-amine

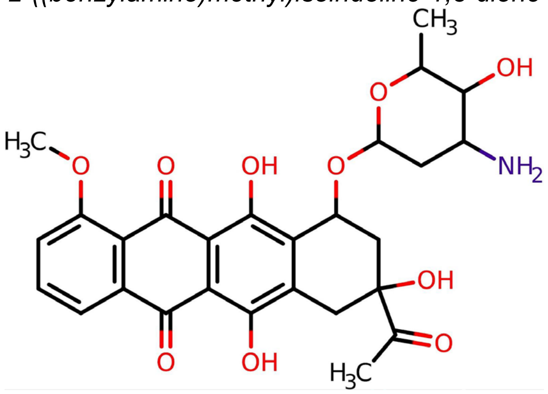

i) Danuorobicin
rac-(8R,10R)-8-acetyl-10-(((2S,4R,5R,6R)-4-amino-5-hydroxy-6-methyltetrahydro-2H-pyran-2-yl)oxy)-6,8,11-trihydroxy-1-methoxy-7,8,9,10-tetrahydrotetracene-5,12-dione

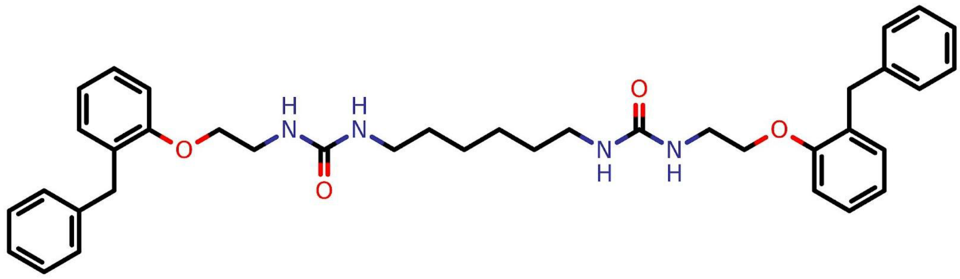

j) AN-153I103354
1,1'-(hexane-1,6-diyl)bis(3-(2-(2-benzylphenoxy)ethyl)urea)

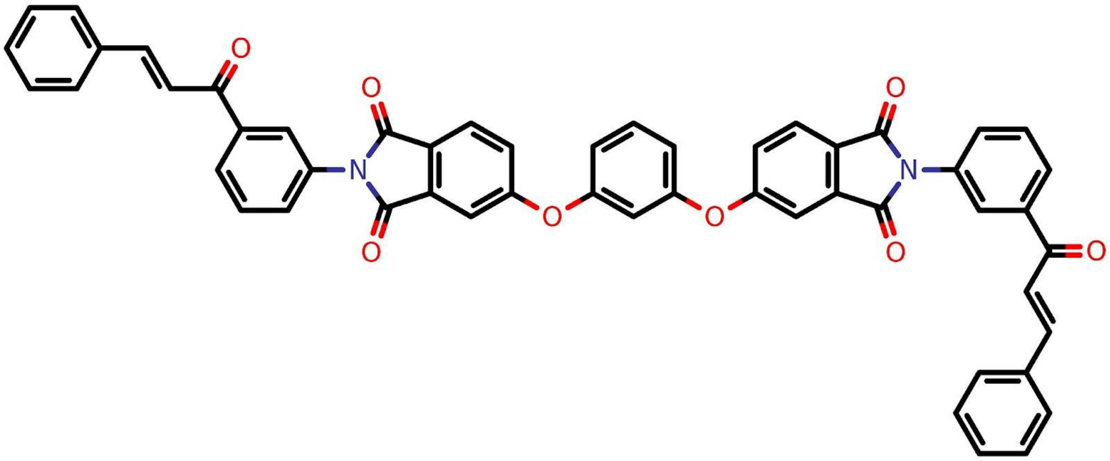

k) AN-153I101592
5,5'-(1,3-phenylenebis(oxy))bis(2-(3-cinnamoylphenyl)isoindoline-1,3-dione)

Figure S1. (continuation) Chemical structure and names of the selected compounds by the Virtual high-throughput screening.

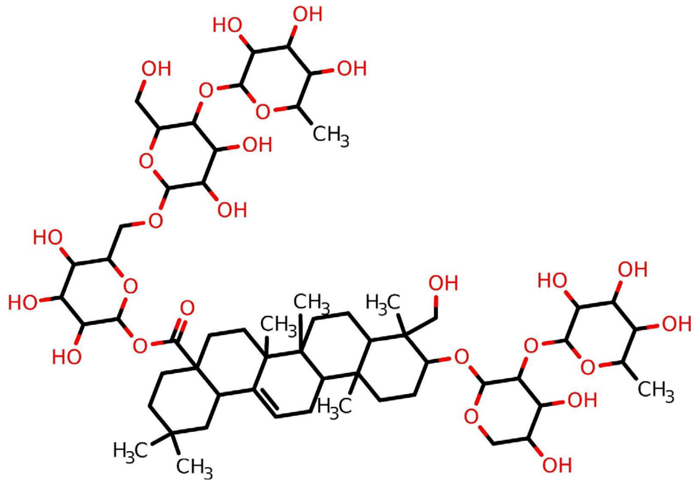

l) Hederacoside C:

[(2S,3R,4S,5S,6R)-6-[[(2R,3R,4R,5S,6R)-3,4-dihydroxy-6-(hydroxymethyl)-5-[(2S,3R,4R,5R,6S)-3,4,5-trihydroxy-6-methyloxan-2-yl]oxyoxan-2-yl]oxymethyl]-3,4,5-trihydroxyoxan-2-yl] (4aS,6aR,6aS,6bR,8aR,9R,10S,12aR,14bS)-10-[(2S,3R,4S,5S)-4,5-dihydroxy-3-[(2S,3R,4R,5R,6S)-3,4,5-trihydroxy-6-methyloxan-2-yl]oxyoxan-2-yl]oxy-9-(hydroxymethyl)-2,2,6a,6b,9,12a-hexamethyl-1,3,4,5,6,6a,7,8,8a,10,11,12,13,14b-tetradecahydropicene-4a-carboxylate

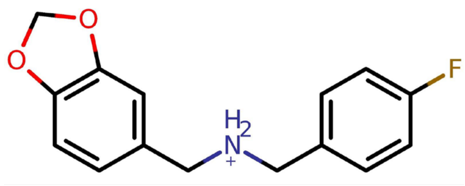

m) AN-153KC12612

1-(benzo[d][1,3]dioxol-5-yl)-N-(4-fluorobenzyl)methanamine

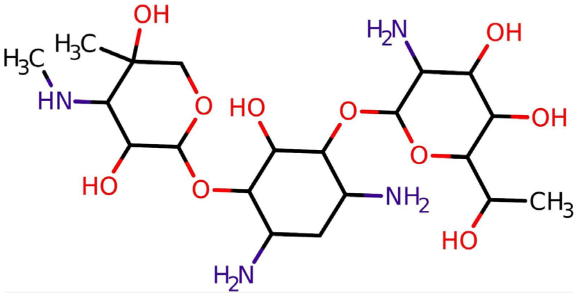

n) Geneticin

(2RS,3SR,4RS,5RS,6SR)-5-amino-6-(((1SR,2RS,4SR,6RS)-4,6-diamino-3-(((3RS,4RS,5RS)-3,5-dihydroxy-5-methyl-4-(methylamino)tetrahydro-2H-pyran-2-yl)oxy)-2-hydroxycyclohexyl)oxy)-2-((S)-1-hydroxyethyl)tetrahydro-2H-pyran-3,4-diol

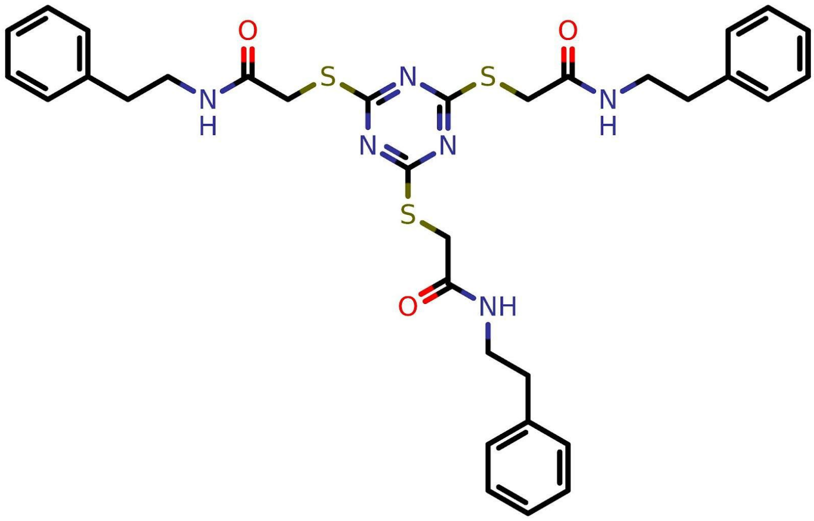

o) AN-153I104845

2,2',2''-((1,3,5-triazine-2,4,6-triyl)tris(sulfanediyl))tris(N-phenethylacetamide)

Figure S1. (continuation) Chemical structure and names of the selected compounds by the Virtual high-throughput screening.

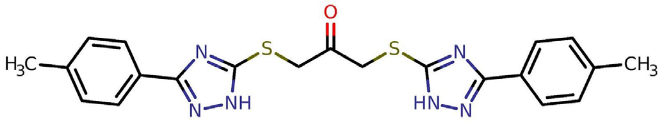

p) AN-153I103073
1,3-bis((3-(p-tolyl)-1H-1,2,4-triazol-5-yl)thio)propan-2-one

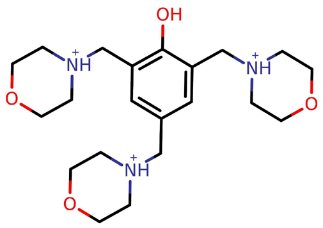

q) AN-153I100720
2,4,6-tris(morpholinomethyl)phenol

Figure S1. (continuation) Chemical structure and names of the selected compounds by the Virtual high-throughput screening.

Section S2. Molecular Dynamics simulations

a) α-amylase **b)** co-crystallized **c)** mode 1 **d)** mode 5

Figure S2. QRcodes with url links for MD simulation movies of α-amylase without drug (**a**), and α-amylase with acarbose (**b** to **d**), at different interaction sites. Water molecules and counter ions were omitted for better visualization.

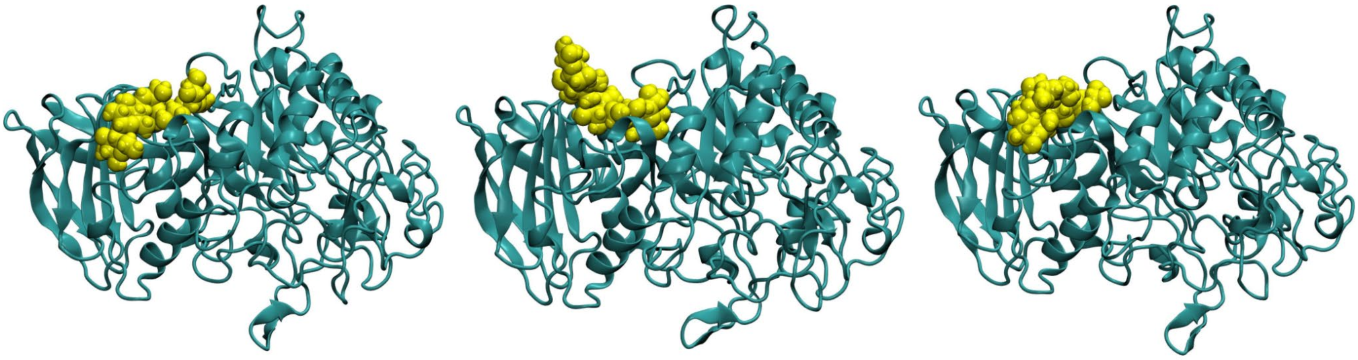

a) 0 ns **b)** 16.4 ns **c)** 131 ns

Figure S3. Snapshots of the simulation of α-amylase and acarbose starting in mode 1 from blind docking.

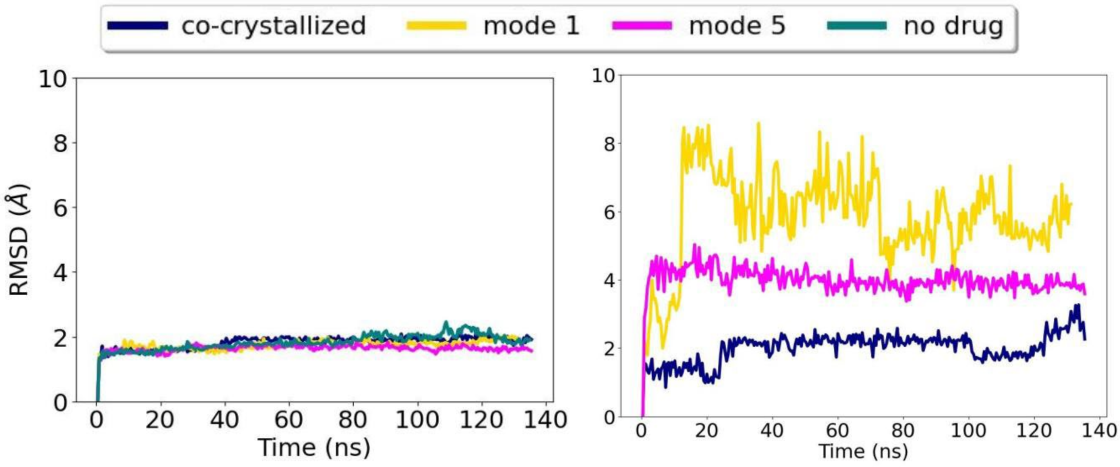

a) enzyme **b)** acarbose

Figure S4. Mass weighted RMSD plots of α-amylase (**a**) and acarbose (**b**) obtained from molecular dynamics. Plots of α-amylase (**a**) refer to: co-crystallized structure, blind docking mode 1, blind docking mode 5, and enzyme without drug. Plots of acarbose (**b**) refer to: co-crystallized structure, blind docking mode 1, and blind docking mode 5.

Section S2.1 Binding free energy calculation

The binding free energy ($\Delta G^{bind,solvated}$, in solution) of the simulated drugs was calculated from the trajectories through Molecular Mechanics/Generalized Born Surface Area (MM-GBSA) method, using the python script *MMPBSA.py*[1] from AmberTools. $\Delta G^{bind,solvated}$ is defined as the difference between complex free energy and the summation of enzyme and drug free energies when unbounded (Eq. 1). In this equation, each solvated term can be obtained as defined by Eq. 2, using single point energy averages (indicated in angle brackets) over the simulated ensemble. Gas-phase energies (E^{gas}) were calculated with molecular mechanics (MM), while the solvation free energy ($\Delta G^{solvation}$) was calculated using the Generalized Born (GB) implicit model.[2] Additionally, rotational and translational entropies are calculated, considering that the system can be described by the rotor rigid model, and the vibrational entropy is derived from the normal mode analysis, so the entropic term (S^{solute}) can be estimated.

$$\Delta G^{bind,solvated} = \Delta G^{complex,solvated} - [\Delta G^{enzyme,solvated} + \Delta G^{drug,solvated}] \quad \textbf{Eq. 1}$$

$$\Delta G^{solvated} \cong \langle \Delta G^{solvation} \rangle + \langle E^{gas} \rangle - T\langle S^{solute} \rangle \quad \textbf{Eq. 2}$$

Moreover, the binding free energies can be decomposed into van der Waals, electrostatic, polar and non-polar solvation energy terms, providing meaningful insights about enzyme-ligand interactions.

Table S1. Average energy values (in kcal/mol) were derived with MM-GBSA analysis over the simulations, and respective standard deviation. Van der Waals energy (**E^{VDW}**), electrostatic energy (**E^{EL}**), polar solvation energy (**E^{GB}**), non-polar solvation energy (**E^{SURF}**), total solvation free energy (**G^{solv}**), total gas-phase free energy (**G^{gas}**), binding free energy in solution (**$\Delta G^{bind,solv}$**).

	**acarbose**		
	**crystallized**	**mode 5**	**mode 1**
**E^{VDW}**	-70.53 ± 5.40	-54.45 ± 4.82	-37.05 ± 9.38
**E^{EL}**	-281.70 ± 20.54	-193.08 ± 15.41	-58.71 ± 20.68
**E^{GB}**	278.49 ± 16.01	202.75 ± 10.88	77.19 ± 16.84
**E^{SURF}**	-10.48 ± 0.44	-8.46 ± 0.30	-5.31 ± 1.16
**G^{gas}**	-352.23 ± 20.23	-247.54 ± 14.35	-95.76 ± 23.30
**G^{solv}**	268.01 ± 15.95	194.29 ± 10.79	71.87 ± 16.36
**$\Delta G^{bind,solvated}$**	-84.23 ± 7.43	-53.25 ± 5.64	-23.89 ± 10.30

[1] Miller III, B. R., McGee Jr, T. D., Swails, J. M., Homeyer, N., Gohlke, H., Roitberg, A. E. MMPBSA. py: an efficient program for end-state free energy calculations. J. Chem. Theory Comput. **2012**, 8(9):3314-3321. https://doi:10.1021/ct300418h
[2] Homeyer, N., Gohlke, H. Free energy calculations by the molecular mechanics Poisson− Boltzmann surface area method. Mol. Inform. **2012**, 31(2):114-122. https://doi:10.1002/minf.201100135

Section S3. Interaction of candidate drugs with α-amylase

Table S2. Definition of labels given to the interactions established between enzyme amino acids and candidate drugs. Geometrical criteria for the presence of the interactions are the default values set by BIOVIA Discovery Studio Visualizer v21.1.

Label	Definition
HB	Hydrogen bond D-H···A, where D is the hydrogen bond donor atom and A the hydrogen bond acceptor atom. Comprises both conventional and Carbon (D = C) hydrogen bonds.
Pi-Donor	Non-classical hydrogen bond, where the hydrogen bond acceptor atom is a pi-system.
Salt Bridge	Interaction between charged groups of opposite sign, combining electrostatic and hydrogen bond interactions.
Alkyl	Hydrophobic interaction between alkyl groups.
Pi-Alkyl	Hydrophobic interaction between a pi-system and an alkyl group.
Pi-Sigma	Hydrophobic interaction between a pi-system and a sigma bond.
Pi-Anion	Hydrophobic interaction between a pi-system and an anion.
Pi-Cation	Hydrophobic interaction between a pi-system and a cation.
Pi-Pi	Hydrophobic interaction between pi-systems. Comprises *Pi-Pi Stacked* and *T-shaped* conformations.
Attractive Charge	Attractive electrostatic interaction between opposite charges.
Positive-Positive	Repulsive electrostatic interaction between positive charges.
Acceptor-Acceptor	Unfavorable clash between hydrogen bond acceptor groups.
Donor-Donor	Unfavorable clash between hydrogen bond donor groups.

Table S3. Results of site-specific and blind docking of potential anti-diabetic compounds with α-amylase using AutoDock Vina. The compound location in site-specific docking was defined by the experimental location of acarbose in α-amylase (1B2Y). Relevant amino acid interactions with α-amylase are specified, and those common to acarbose are highlighted in bold. Docking scores (**DS**) are in kcal/mol. Types of interactions are explained in Table S2. Number of modes in blind docking that correspond to the crystallographic bind-pocket region (**NBC**), number of matching amino acids (**NMAA**) involved in interactions, considering acarbose site specific docking as reference.

**Compound**	**NBC**	**Site-specific docking**			**Blind docking**		
		**DS**	**Amino Acid interaction**	**NMAA**	**DS**	**Amino Acid interaction**	**NMAA**
Acarbose	-	-10.4	*HB*: **ASP-300, ARG-195, GLY-306, GLU-233, GLN-63, HIS-299, HIS-305, TYR-62, THR-163, TYR-151, TRP-59**<br>*Alkyl*: **LEU-165**	12	-8.6	*HB*: ARG-252, ARG-421, GLY-403, TRP-280, HIS-331, PRO-332, PRO-405<br>*Alkyl*: PRO-4	-
Hederacoside C	8	-9.1	*HB*: **ASP-300**, ASP-356, **GLU-233**, **GLY-306**, GLY-304, ASN-352, TRP-357, **HIS-305**<br>*Pi-Alkyl*: **TRP-59**, TRP-58<br>*Alkyl*: **LEU-165**<br>*Pi-Alkyl*+*Pi-Sigma*: **TYR-62**	7	-9.6	*HB*: GLU-282, ARG-421, ASP-402<br>*Pi-Alkyl*: PHE-406<br>*Alkyl*: ARG-398<br>*HB*+*Alkyl*: PRO-332	0
AN-153I104845	8	-8.1	*Pi-Alkyl*: ALA-307, ALA-198, LYS-200<br>*HB*: **GLN-63**, **GLY-306, GLU-233**<br>*Pi-Pi*: **TYR-62**<br>*Pi-Alkyl*+*Pi-Sigma*: ILE-235<br>*Pi-Pi*+*Pi-Sulfur*: **HIS-305**:<br>*Pi-Sulfur*+*HB*: **TRP-59**<br>*Pi-Pi*+*Pi-Cation*+*Pi-Donor*: HIS-201	6	-7.4	*HB*: GLU-240, **HIS-305**, **THR-163**, **ASP-300**, ASP-197<br>*Pi-Sigma*: LEU-162<br>*Pi-Alkyl*: **LEU-165**<br>*Pi-Sulfur*+*Pi-Cation*: HIS-201	4
AN-153I105594	7	-10.8	*Pi-Alkyl*: LEU-162, **LEU-165, HIS-305, TYR-151**<br>*Alkyl*: LYS-200<br>*Pi-Pi*: **TYR-62**<br>*HB*+*Alkyl*: HIS-201<br>*Alkyl*+*Pi-Alkyl*: ILE-235<br>*Pi-Alkyl*+*Pi-Pi*: **TRP-59**	5	-10.3	*Pi-Alkyl*: **TRP-59**, HIS-101, ALA-198, LEU-162, **HIS-305**<br>*Alkyl*: **LEU-165**<br>*Pi-Sigma:* ILE-235<br>*Pi-Cation*+*Pi-Pi*: HIS-201<br>*Pi-Alkyl*+*HB*: LYS-200<br>*Pi-Alkyl*+*Pi-Pi*: **TYR-151**	4
AN-153I104161	7	-8.1	*Pi-Alkyl:* ALA-198, LYS-200<br>*HB*: HIS-101<br>*Pi-Sigma*: ILE-235<br>*Salt Bridge*: **GLU-233**<br>*Attractive Charge*: **ASP-300**<br>*Pi-Pi*: **TYR-62**<br>*Pi-Cation*+*Pi-P*: HIS-201<br>*HB*+*Attractive Charge*+*Pi-Anion:* ASP-197	3	-7.7	*Pi-Alkyl:* LEU-162, LYS-200<br>*Pi-Sigma*: ILE-235<br>*Attractive Charge*: ASP-197<br>*HB*: **GLY-306**<br>*Attractive Charge*+*Pi-Anion:* **ASP-300**<br>*Pi-Cation*+*Pi-Pi*: HIS-201<br>*Salt Bridge*+*Charge Attractive:* **GLU-233**	3
Diprotin A	7	-7.0	*HB*: **HIS-299**, ASP-197, **GLU-233**<br>*Alkyl*: ALA-198, LEU-162<br>*Pi-Alkyl*: **TRP-59**, **HIS-305**	4	-6.7	*HB*: **GLN-63**<br>*Pi-Sigma*: **TRP-59**<br>*Alkyl*: ILE-235, ALA-198 LEU-162<br>*Pi-Alkyl:* **TYR-62,** HIS-201, **TRP-59**	4
Daunorubicin	6	-9.3	*Pi-Alkyl*: LYS-200, ALA-198<br>*HB*: **THR-163**<br>*Acceptor-Acceptor*: **GLU-233**:<br>*Pi-Sigma*: LEU-162<br>*HB*+*Pi-Sigma*: **HIS-305**<br>*Alkyl*+*Pi-Sigma*: ILE-235<br>*Pi-Cation*+*Pi-Pi*: HIS-201	3	-8.8	*HB*: **GLN-63**, HIS-101<br>*Alkyl:* LEU-162<br>*Pi-Alkyl*+*Pi-Pi:* **TRP-59**	2
AN-153I101592	5	-10.6	*Pi-Cation*: **HIS-305**, HIS-201<br>*Pi-Alkyl*: ALA-307<br>*Pi-Pi*: **TYR-151**<br>*Pi-Alkyl*+*Pi-Sigma*: LEU-162	2	-13.3	*HB*: HIS-201, **GLN-63**<br>*Pi-Alkyl*: LEU-162, VAL-107<br>*Pi-Pi*: **TYR-62, TRP-59**<br>*Pi-Anion*: ASP-197<br>*Pi-Sigma*: ILE-51<br>*HB*+*Pi-Pi*: **TYR-151**<br>*Pi-Alkyl*+*Pi-Sigma*: ILE-235	4

Table S3. (Continuation) Results of site-specific and blind docking of potential anti-diabetic compounds with α-amylase using AutoDock Vina. The compound location in site-specific docking was defined by the experimental location of acarbose in α-amylase (1B2Y). Relevant amino acid interactions with α-amylase are specified, and those common to acarbose are highlighted in bold. Docking scores (**DS**) are in kcal/mol. Types of interactions are explained in Table S2. Number of modes in blind docking that correspond to the crystallographic bind-pocket region (**NBC**), number of matching amino acids (**NMAA**) involved in interactions, considering acarbose site specific docking as reference.

Compound	NBC	Site-specific docking			Blind docking		
		DS	Amino Acid interaction	NMAA	DS	Amino Acid interaction	NMAA
AN-153I100720	5	-6.8	Attractive Charge: ASP-356<br>Pi-Pi: **TRP-59**<br>Donor-Donor: **GLN-63**	2	-6.6	HB: **ASP-300**, **THR-163**, **GLN-63**<br>Positive-Positive: **HIS-305**<br>HB+Pi-Pi+Pi-Cation: **TRP-59**	5
Geneticin	4	-7.8	HB: ASP-197, **ASP-300**, **GLU-233**<br>Alkyl: **LEU-165**<br>Pi-Alkyl: **HIS-305**<br>HB+Pi-Alkyl: HIS-101<br>Pi-Alkyl+Pi-Sigma+HB: **TRP-59**	5	-7.7	HB: **GLU-233**, ASP-197, **TYR-151, ASP-300**<br>Alkyl: LEU-162, **LEU-165**<br>Donor-Donor: **GLN-63**, **GLY-306**	6
Octopamine	3	-5.7	HB: ASP197, **ASP-300**, **GLU-233**<br>Pi-Pi: **TYR-62**	3	-5.0	HB: **ASP-300**, ASP-197, **TRP-59**<br>Pi-Pi: **TYR-62**<br>Donor-Donor: **ARG-195**	4
Kynuramine	3	-5.6	HB: ASP-197, **GLU-233**<br>Pi-Anion: **ASP-300**<br>Pi-Pi: **TYR-62**<br>Donor-Donor: **ARG-195**	4	-5.5	Pi-Pi+HB: **TYR-62**	1
AN-153I100678	3	-5.0	HB: GLY-309, GLN302, ILE-312, ALA-310, ASN-301<br>Positive-Positive: ARG-346, ARG-267	0	-4.9	HB: GLY-309, GLN-302, ILE312, ALA-310, ASN-301<br>Salt Bridge: ASP-317<br>Positive-Positive: ARG-346	0
AN-153I103073	2	-7.0	Pi-Alkyl: TRP-58, **TYR-62**<br>HB: **GLN-63**, **THR-163**<br>Pi-Pi: **TRP-59**<br>Alkyl+Pi-Alkyl: LEU-162	4	-7.9	Pi-Alkyl: **TYR-62**, TRP-58, **LEU-165**, VAL-107, ILE-51<br>HB: **GLN-63**<br>Pi-Pi: **TRP-59**<br>Pi-Alkyl+Pi-Donor: TYR-52	4
AN-153I103354	1	-7.2	HB: ASP-197, **GLU-233**, **GLY-306, ASP-300**<br>Alkyl: ALA-198, ILE-235<br>Pi-Alkyl: **LEU-165**, **TYR-151**<br>Pi-Pi: **TRP-59**<br>Alkyl+Pi-Alkyl: LEU-162<br>Pi-Pi+Pi-Cation: **HIS-305**	7	-5.4	HB: ARG-252, SER-289	0
Hematoporphyrin	0	-9.2	HB: ASP-197, **HIS-299**, HIS-201, **GLU-233**<br>Pi-Alkyl: **TYR-151**, **HIS-305**<br>Alkyl: ILE-235<br>Donor-Donor: **THR-163, ARG-195**<br>Pi-Alkyl+Pi-Sigma: **TRP-59, TYR-62**<br>Pi-Sigma+Alkyl: LEU-162	8	-7.0	HB: THR-6, ASN-5, ARG-10<br>Pi-Alkyl: PHE-335<br>Pi-Anion: ASP-402<br>Pi-Cation: ARG-252	0
AN-153KC12612	0	-7.8	Pi-Pi: **TYR-151**, TRP-58,**TYR-62**<br>Pi-Alkyl: LEU-162, ALA-198<br>Salt Bridge: **GLU-233**<br>Pi-Sigma: ILE-235<br>Pi-Pi+Pi-Donor: HIS-201<br>HB+Attractive Charge: ASP-197<br>Attractive Charge+Pi-Anion: **ASP-300**<br>Pi-Alkyl+HB: LYS-200	4	-5.6	Pi-Donor: SER-132<br>Attractive Charge: ASP-135<br>HB: LYS-172, PRO-130<br>Pi-Pi: TYR-174	0
